# Audit of Patient Deaths and Incident Reporting in the Acute Old Age Psychiatry Inpatient Wards at the Royal Edinburgh Hospital, NHS Lothian, Scotland

**DOI:** 10.1192/bjo.2026.11735

**Published:** 2026-06-30

**Authors:** Catherine-Anne Convery, Carol-Anne Sherriff, Carrie Coull

**Affiliations:** Royal Edinburgh Hospital, NHS Lothian, Edinburgh, United Kingdom

## Abstract

**Aims:**

This audit focused on the recording and reporting of patient deaths across the Acute Old Age Psychiatry wards at the Royal Edinburgh Hospital (REH), Scotland. All inpatient deaths should be reported electronically via the NHS Lothian health board ‘DATIX’ incident reporting system. A patient death is recorded as ‘expected’ or‘unexpected’. This audit aimed to identify how many patient deaths had occurred over a 2 year period and if they were appropriately reported via ‘DATIX’.

**Methods:**

In January 2025 retrospective data was gathered for the review period from January 2023 to December 2024.

Following a patient death, a physical Medical Certificate of Cause of Death (MCCD) was issued by medical staff. The MCCD log books are held on the Acute Old Age Psychiatry wards at REH. Each log book entry was reviewed and the number of deaths were recorded.

Electronic data from the ‘DATIX’ reporting system was gathered by the Assurance and Improvement Team for the REH, who are involved in the review of Significant Adverse Events.

The information from the MCCD log books and ‘DATIX’ system were cross-referenced.

**Results:**

A total of 14 patients died whilst an inpatient during the review period. These were all due to a primary medical condition and not primarily resulting from a psychiatric disorder.13 MCCD were issued. 1 MCCD could not be issued due to legal reasons. The patient deaths occurred on 3 of the 4 wards.

‘DATIX’ data identified that 7 deaths were reported; all 3 ‘unexpected’ deaths and 4 ‘expected’ deaths.

Therefore, 50% of the patient deaths occurring on the Acute Old Age Psychiatry wards at REH were not reported via the ‘DATIX’ incident reporting system.

**Conclusion:**

This audit identified the number of patient deaths that occurred during the 2 year review period on the Acute Old Age Psychiatry wards at REH and elucidated that not all patient deaths were being reported to the ‘DATIX’ incident system. In particular, ‘expected’ deaths were not reported consistently. This meant data held by the Assurance and Improvement Team was not accurate and highlighted that clinical teams were not aware of the need to report every inpatient death. Following this audit, issuing of the MCCD at REH became electronic. Senior management also ensured all clinical teams were informed of the requirement to report all deaths via ‘DATIX’. Future audit is required.

